# An elusive chest coin in an african child: a pleural fibroma's long, tortuous path to freedom

**DOI:** 10.11604/pamj.2013.14.16.1874

**Published:** 2013-01-10

**Authors:** Ademola Adegoke Aremu, Olusola Adetunji Oyedeji, Christianah Mopelola Asaleye, Victor Adebayo Adetiloye

**Affiliations:** 1 Radiology Department, Ladoke Akintola University Teaching Hospital, Osogbo, Osun State, Nigeria; 2 Paediatric Department, Ladoke Akintola University Teaching Hospital, Osogbo, Osun State, Nigeria; 3 Radiology Department, Obafemi, Awolowo University Teaching Hospital, Ile-Ife, Osun State, Nigeria

**Keywords:** Pleural fibroma, rare, teenager, scarce resources, diagnostic difficulty

## Abstract

Fibrous tumour of the pleural is rare and controversial tumor. Most of the reported cases is adults and the elderly. This case presentation is a solitary fibrous tumour in a fifteen year old girl, which to the best of our knowledge is the youngest report, who was sent for a psychiatric evaluation due to persistent complaint of “movement” in her chest, later referred to a tuberculosis clinic because of a chest radiograph report of loculated pleural effusion likely secondary to tuberculosis. She eventually had a chest computerized tomography and subsequent resection of the lesion. Histology confirmed the computerized tomography diagnosis of solitary fibrous tumour and there was no recurrence five years after excision. This report highlights the difficulty often encountered in developing countries where clinicians solely rely on clinical acumen for diagnosis and treatment due to poor patients’ financial status and scarcely available diagnostic resources.

## Introduction

Solitary fibrous tumours (SFT) of the pleural are rare, controversial tumour accounting for less than 5% of all neoplasm involving the pleural [1, 2]. They present a diagnostic dilemma and can easily be missed even in an environment with adequate clinicoradiologic work up [2]. It affects women and men of all ages with the mean age of 51 years [3] though all the reported cases are adults and elderly, our patient is a teenager.

SFT are different from the commoner pleural tumor, diffuse mesothelioma in that they are not related to asbestos’ exposure, they have good prognosis and are not aggressive [4]. They mostly arise from the visceral pleural (80% of cases) but can also be derived from parietal pleural; other serosal membranes like peritoneum, pericardium non serosal sites such as the lung parenchyma, the nose and paranasal sinuses [2].

They are often misdiagnosed because of their various thoraxic and extrathoraxic manifestations; and also wide variation in sizes and behaviour.

The objective of this case report is to call the attention of physicians practising in the third world where fund and availability of diagnostic equipment are essential consideration in patients management, to the misleading, vague and often multi-systemic presentation of this rare but curable pathology.

## Patient and observation

OT, a 15 year- old final year secondary school student was taken to the village health centre by the school principal because of a persistent discomfort from a moving object in the chest that prevented her from attending the secondary school leaving certificate examination. The symptoms had been on for about a year but just got worsened. There was no other associated symptom nor abnormal finding on examination though Xray machine was not available at the centre. He reassured and placed on multivitamins and analgesic but reffered to a psychiatrist after six weeks because there was no improvement.

The psychiatrist assessed and made a diagnosis of generalized anxiety disorder, requested for a chest radiograph and full blood count. She defaulted from the clinic and was not able to do the tests for “financial reasons”. She re-presented about fourteen months later with additional six months history of recurrent, productive cough, fever, weight loss and bilaterally swollen nail-bedth.

**Figure 1:**
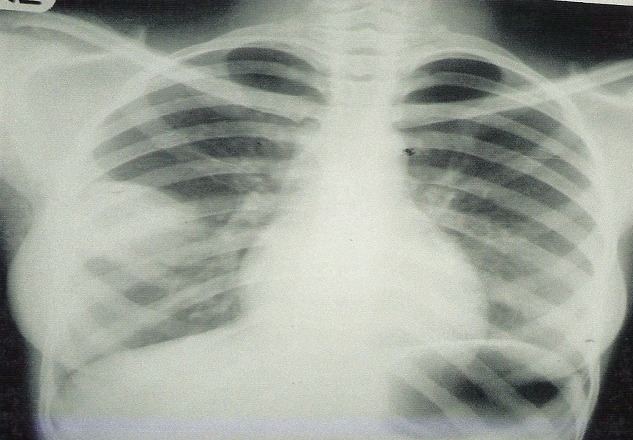
Frontal chest radiograph showing a fairly well marginated, rounded homogenous opacity in the lateral aspect of the right middle lung zone

**Figure 2:**
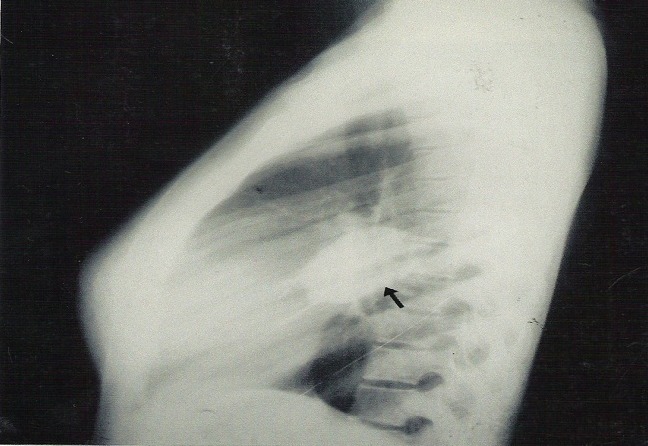
Lateral chest radiograph showing a well defined oval shaped homogenous opacity in the fissure

Clinical examination revealed a young girl, mildly pale, anicteric, febrile but not in any obvious respiratory distress with grade IV finger clubbing. She was not tachypnoiec but there was stone dullness and reduced air entry in the right middle lung zone. She was financially assisted to do Chest radiograph, Full blood count(FBC), fasting blood sugar(FBS) and sputum alcohol acid fast bacilli(AAFB). The FBC and FBS were normal; the sputum AAFB was negative on three occasions but the chest radiograph (Figure 1, Figure 2) showed a rounded, well-defined- lobulated peripherally located mass in the (RT) middle lung zone, measuring about 6x5cm and forming an acute angle with the lateral chest wall. There was no calcification, satellite pulmonary lesion, periosteal reaction, or bony erosion.

A diagnosis solitary pulmonary nodule “chest coin”: encysted pleural effusion secondary to tuberculosis was made; to rule out pleural fibroma and lipoma. She couldn't afford the requested Chest CT and was placed on the free anti tuberculosis regime for few months.

Evaluation clinically and with radiograph showed there was no improvement; fund was sourced for her for a chest CT. Enhanced CT scan revealed a well defined rounded, peripherally located soft tissue density mass in the right middle lung zone on the scannogram. Serial cuts showed a fairly homogenous, well defined soft tissue density mass with no calcific nor cystic area within it. There was minimal enhancement post intravenous contrast. There was no pleural effusion nor invasion of the surrounding lung tissue, mediastinum and ribs. The diagnosis of pleural fibrous tumor was confirmed.

At surgery, a fibrocystic mass of about 6x7cm with stalk on the (Rt) lower lung lobe and adhering to the lateral chest wall was found.

Histology confirmed a diagnosis of benign solitary fibrous tumor. The patient was discharged two weeks after surgery. The post op chest radiograph did not reveal any residual mass, initial six monthly and later yearly review for the past five years did not show any evidence of recurrence.

## Discussion

The first solitary fibrous tumor was described by Wagner in his article “Das Tuber Kelahnliche Lymphadenom”. In 1931, Klemperer and Rabin discovered a diffuse type of tumor that arose from mesothelial layer as different from a localized type arising from the sub mesothelial fibrous connective tissue. In 1952, Clagett et al used the term localized fibrous mesothelioma to distinguish these usually benign tumors from more common asbestos- related, malignant mesothelioma [5].

Primary tumor of the pleural can be divided into two major categories: diffuse and solitary [3]. The diffuse primary pleural tumor are mesotheliomas- they are commoner than solitary pleural tumors, arise from mesothelial tissue, associated with asbestos exposure and almost always have a fatal course [6]. The presented case had the less common solitary primary tumor of he pleura and was not associated with asbestos expoxure.

SFTs have been known by variety of names due to their clinical course and controversies surrounding their histogenesis. Benign mesothelioma, localized mesothelioma, localized fibrous tumor of the pleural and subpleural fibrous tumor are some of the previous terminologies [3, 7, 8]. They are now called solitary fibrous tumors because they are of mesenchymal rather than mesothelial origin [9]. They are not related to mesothelioma, and asbestos exposure, are mostly benign in greater than 80% of cases. The histology report in the patient presented suggested a benign lesion.

Due to their mesenchymal histiogenesis, they can be found not only in the pleural space but also in the lung, pericardium, mediastinum, upper respiratory tract peritoneum, liver, thyroid and orbit [2, 9]. In this case report, the solitary fibrous tumor is located in the pleura though had its stalk attached to the right lower lobe.

Only about six hundred cases had been reported previously [4, 10]; this is the first case seen by the authors with not less than hundred cumulative years of clinical practice and the first reported case from Nigeria to the best of our knowledge. About 13% of reported cases had an aggressive clinical behaviour with local infiltration, local recurrence and rarely distant metastases. The remaining 87% had benign clinical behaviour and radial resection was sufficient treatment [7].

Localized fibrous tumours are comprised of haphazardly arranged fascicles of spindle cells, separated by variable amounts of collagen [3]. They are found commonly in the dependent portion of the thorax with the size ranging between 1-39cm in diameter [3]. They usually displace (if large) rather than invade adjacent structures and may show a change in shape and location with changes in position or respiration because of presence of vascular pedicle in 30-50% of the patients. The presented case was however not large enough to displace adjacent structures.

Patients usually present with cough, shortness of breath, chest pain, sensation of a mass moving within the chest and dyspnoea. These features are seen in about 40% of cases while 54% are asymptomatic [5]. Our patient only had the sensation of a mass moving in the chest initially but unfortunately Xray machine was not available and the clinician was not suspecting pleural fibroma. There is a need for awareness and high index of suspicion amongst clinicians. Extrathoraxic manifestations like hypoglycemia, digital clubbing, hypertrophic pulmonary osteoarthropathy, arthritic pain and fever are also possible [5].

Though both solitary fibrous tumour and mesothelioma can grow to massive proportion with life threatening compression of intrathoraxic structures; clubbing, hypertrophy osteoarthropathy and hypoglycaemia are not typically seen with mesothelioma. Therefore the combination of one of these paraneoplastic syndromes with pleural mass should suggest a diagnosis of solitary fibrous tumor [3].

Hypoglycaemia is said to be caused by tumor production of insulin- like growth factor II [3]. Unrecognized solitary fibrous tumor had resulted into hypoglycaemic coma & death [3].

Though lung cancer with brain metastases is a possibility in patients presenting with chest tumor and altered mental states, solitary fibrous tumor (with hypoglycaemia) should also be considered.

Paraneoplastic tumor are noticed to resolve following surgical resection of the solitary fibrous tumor [7]. There was clinical evidence of gradual reduction in the finger clubbing in this patient at six months and became innocures by two years postop.

Radiological diagnosis is usually difficult though chest radiograph and Chest CT are the imaging modalties of choice, they lack specificity and cannot differentiate benign and malignant fibrous tumor [9]. Fluorodeoxyglucose PET scan, though not available, will differentiate fibrous tumor from malignant mesothelioma due to significant increase in uptake by the latter [9]. CT guided fine needle biopsy is often not reliable and at best have 45% accuracy [10].

Magnetic Resonance Imaging (MRI) is useful in assessing the relationship of the tumor to neighboring structures and evaluate respectability while angiography may be relevant in large vascular variants with possibility of a preoperative percutaneous embolization [2]. Ultrasound may show a well defined hyperechoic mass with lobulated contours forming obtuse angles where it meets the chest wall [3]. Attempts at scanning this patient did not yield useful information. Histologic and Immunohistochemical (Anti-CD34 antibody) analysis of sample obtained by transthoraxic Tru-cut biopsy give a confident preoperative diagnosis and exclude differential diagnosis like mesothelioma, fibrosarcoma, fibrous histiocytoma, fibromatosis, synovial sarcomas [3, 9].

Video assisted thoracoscopic surgery(VATS) and or open technique is/are the treatment of choice. The prognosis depends firstly on the resectability of the tumor, secondly on its size, and then, in decreasing order of importance, on the mitotic count, polymorphism, and necrosis within the tumor [2, 3]. Extended resection such as lobectomies, chest wall resection etc may be needed [3].

This case had a good prognosis because of the small size of the tumor, its resectability and histological assessment.

Surgical resection is curative in most cases while recurrence is possible in very few cases though the role of adjuvant therapy is yet to be established [2]. However, long term follow up and curative re-resection is necessary because of late recurrences [2].

## Conclusion

We have discussed a rare case of pleural fibroma in a teenager, highlighted the difficulty posed by its vague symptoms and scarce resources in our environment.
